# A tumor microenvironment-responsive micelle co-delivered radiosensitizer Dbait and doxorubicin for the collaborative chemo-radiotherapy of glioblastoma

**DOI:** 10.1080/10717544.2022.2108937

**Published:** 2022-08-16

**Authors:** Shuyue Zhang, Xiuxiu Jiao, Michal Heger, Shen Gao, Mei He, Nan Xu, Jigang Zhang, Mingjian Zhang, Yuan Yu, Baoyue Ding, Xueying Ding

**Affiliations:** a Shanghai General Hospital, Shanghai Jiao Tong University School of Medicine, Shanghai, China; b Department of Pharmacy, Shanghai 9th People's Hospital, Shanghai Jiao Tong University School of Medicine, Shanghai, China; c Jiaxing Key Laboratory for Photonanomedicine and Experimental Therapeutics, Department of Pharmaceutics, College of Medicine, Jiaxing University, Jiaxing, China; d Department of Pharmaceutical Science, School of Pharmacy, Naval Medical University, Shanghai, China

**Keywords:** Glioblastoma, radiosensitization, targeted nanotherapeutics, mic-roenvironment-responsive, chemo-radiotherapy

## Abstract

Glioblastoma is rather recalcitrant to existing therapies and effective interventions are needed. Here we report a novel microenvironment-responsive micellar system (ch-K5(s-s)R8-An) for the co-delivery of the radiosensitizer Dbait and the chemotherapeutic doxorubicin (DOX) to glioblastoma. Accordingly, the ch-K5(s-s)R8-An/(Dbait-DOX) micelles plus radiotherapy (RT) treatment resulted in a high degree of apoptosis and DNA damage, which significantly reduced cell viability and proliferation capacity of U251 cells to 64.0% and 16.3%, respectively. The angiopep-2-modified micelles exhibited substantial accumulation in brain-localized U251 glioblastoma xenografts in mice compared to angiopep-2-lacking micelles. The ch-K5(s-s)R8-An/(Dbait-DOX) + RT treatment group exhibited the smallest tumor size and most profound tumor tissue injury in orthotopic U251 tumors, leading to an increase in median survival time of U251 tumor-bearing mice from 26 days to 56 days. The ch-K5(s-s)R8-An/(Dbait-DOX) micelles can be targeted to brain-localized U251 tumor xenografts and sensitize the tumor to chemotherapy and radiotherapy, thereby overcoming the inherent therapeutic challenges associated with malignant glioblastoma.

## Graphical Abstract

**Figure:**
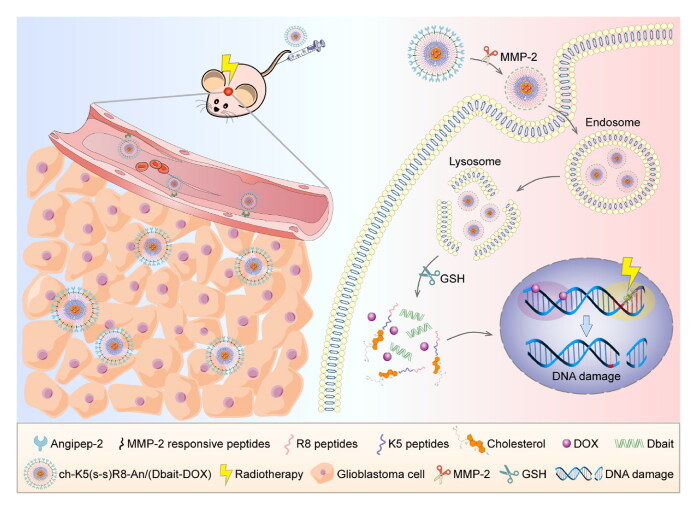

## 1. Introduction

In adults, glioblastoma is the most common primary malignant brain tumor and its prognosis is particularly poor (Ostrom et al., 2014). Standard treatment includes surgery, radiotherapy (RT), and/or chemotherapy. However, therapeutic efficacy against glioblastoma has remained poor and the malignancy typically recurs within 7 months (Siegel et al., 2020), rendering glioblastoma a life-threatening problem even in patients whose treatment was initially successful (Siegel et al., 2020). The most pivotal problems of glioblastoma treatment are inherent RT resistance (Dutreix et al., 2010; Zhang et al., 2019), tolerance to chemotherapy drugs (Fan et al., 2014; Le Rhun et al., 2019), and low drug permeability due to the blood-brain barrier and the blood-brain tumor barrier (Zhao et al., 2017; Arvanitis et al., 2020).

These obstacles and the severe adverse effects associated with conventional glioblastoma therapies have created a medical need to develop new, safer, and more effective interventions for glioblastoma (Tang et al., 2019). To improve therapeutic outcomes, combination regimens such as concurrent chemo-radiotherapy have gained traction in basic research and clinical trials (Neoptolemos et al., 2004; Song et al., 2017), addressing both the primary tumor and the metastatic lesions outside of the irradiation zone. The synergistic effects of combination therapies have been demonstrated for glioblastoma and other cancer types (Gomez et al., 2016; Zhang et al., 2017).

The treatment efficacy of chemo-radiotherapy can be expanded by pharmacologically rendering tumor tissue more susceptible to RT. To that end, stabilized DNA molecules (Dbait) were designed to mimic DNA double-strand breaks and give off a ‘false’ DNA damage signal, ultimately inhibiting the recruitment of proteins involved in double-strand break repair and forcing cells into apoptosis (Quanz et al., 2009; Biau et al., 2014). Recent reports have shown that the use of Dbait as radiosensitizers in combination with RT is effective for the treatment of glioblastoma, head and neck squamous cell carcinoma, skin melanoma, and colorectal cancer metastases, amongst others (Devun et al., 2014; Biau et al., 2016; Liu et al., 2017).

We previously developed a blood-brain barrier-targeting and glioblastoma cell-targeting micellar system (ch-K5(s-s)R8-An) to deliver Dbait into glioblastoma cells and make these cells more sensitive to RT (Jiao et al., 2019). In this study, Dbait and the chemotherapeutic doxorubicin (DOX) were further co-encapsulated into ch-K5(s-s)R8-An micelles on the basis of our previous work, which aimed to mount a blitz attack on the tumor cell’s DNA infrastructure by damaging DNA with RT and concomitantly ceasing macromolecular biosynthesis and topoisomerase II progression with DOX (Pommier et al., 2010; Tacar et al., 2013). Such synergistic mechanism induced maximum apoptotic signaling and consequent cell death in cultured human malignant glioblastoma (U251) cells and *in situ* glioblastoma xenografts in mice.

**Scheme 1.**
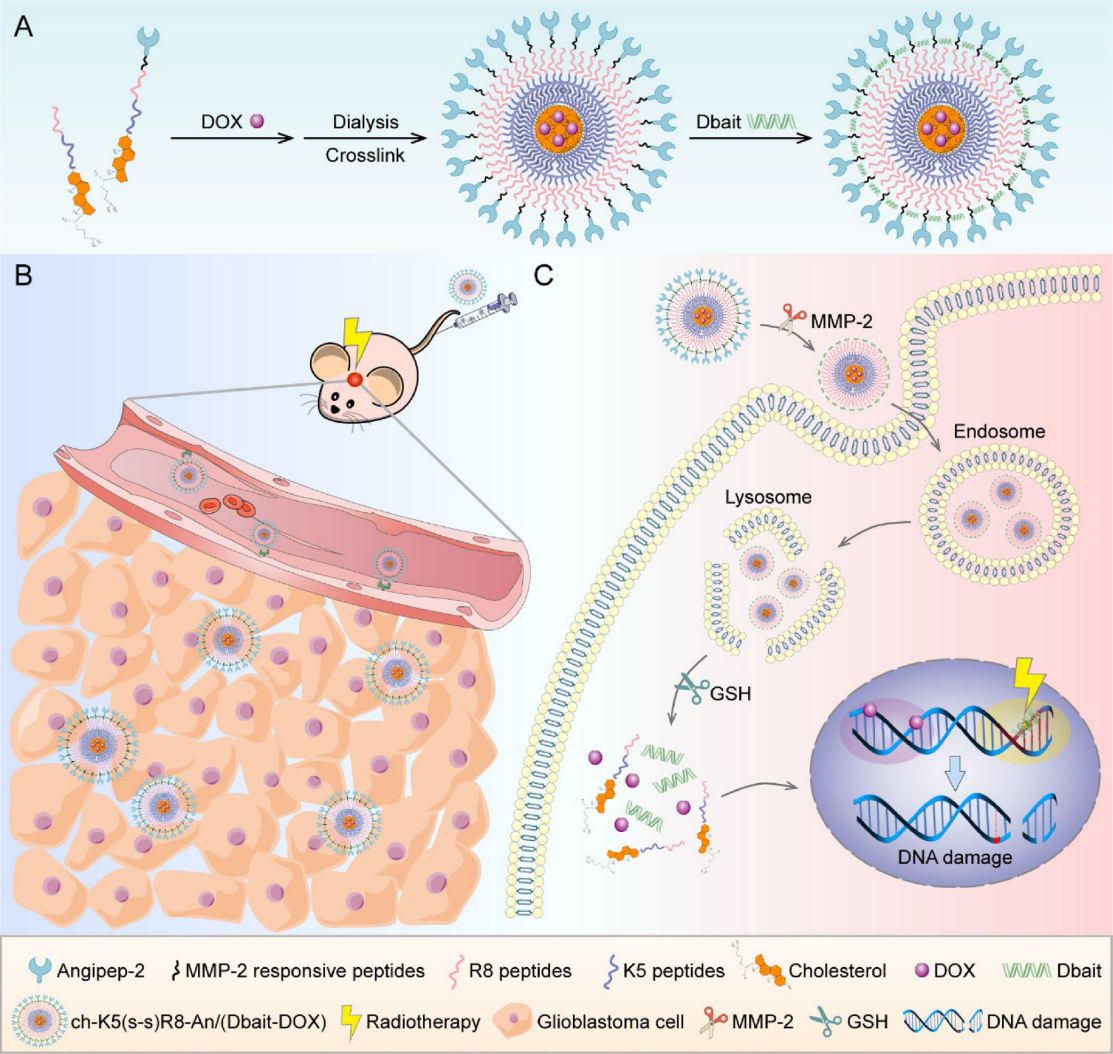
The preparation and anti-glioblastoma mechanism of the Dbait and DOX co-loaded micellar system. (A) The preparation of ch-K5(s-s)R8-An/(Dbait-DOX) micelles. (B) The application of ch-K5(s-s)R8-An/(Dbait-DOX) for chemo-radiotherapy in orthotopic glioblastoma-bearing nude mice. (C) The synergistic mechanism of ch-K5(s-s)R8-An/(Dbait-DOX) as chemo-radiosensitizer.

To this end, ch-K5(s-s)R8-An/(Dbait-DOX) micelles were developed as a multi-step co-delivery system (Scheme 1). Step 1, the angiopep-2 (An) that is conjugated to the micelle outer shell serves to target the micelles to the blood-brain barrier (Huang et al., 2013; Ruan et al., 2015; Feng et al., 2017). Step 2, the enzymatically cleavable linkers (matrix metalloproteinase 2 [MMP-2] responsive peptides) that are attached to the cell penetration-enhancing R8 peptide in the middle shell ensure the glioblastoma cell internalization following MMP-2 cleavage in the tumor microenvironment (Cui et al., 2016). Step 3, the K5-cholesterol anchors comprise the inner shell and are cross-linked *via* disulfide bridges for high drug loading capacity and triggered drug release under the reductive environment in the cytosol of tumor cells (Yang et al., 2018). The collaborative anti-glioblastoma effect of ch-K5(s-s)R8-An/(Dbait-DOX) plus RT was investigated *in vitro* and *in vivo*.

## 2. Materials and methods

### 2.1. Materials

The cholesterol-KKKKKRRRRRRRR (ch-K5R8) peptides and cholesterol-KKKKKRRRRRRRR-Angiopep-2 (ch-K5R8-An) peptides were designed by our group and then synthesized by Ontores Biotechnologies (Shanghai, China). Other materials used in this study are listed in the Supplementary Material.

### 2.2. Cell lines and animals

Information on the human malignant glioblastoma cell line U251 and male Balb/c nude mice are described in the Supplementary Material. All animal experiments were performed according to the *National Institute of Health Guidelines for the Care and Use of Laboratory Animals* (8th edition) and institutional guidelines under the approval of the institutional review board of Shanghai Jiao Tong University. Animal caring details are listed in Supplementary Material.

### 2.3. Micelle preparation and characterization

Using the membrane dialysis method, ch-K5(s-s)R8 micelles, ch-K5(s-s)R8-An micelles, ch-K5(s-s)R8-An/DOX micelles, ch-K5(s-s)R8-An/Dbait micelles, and ch-K5(s-s)R8-An/(Dbait-DOX) micelles were prepared. The particle size, polydispersity index (PDI), and zeta potential of these micelles were measured by dynamic light scattering and electrophoretic mobility analysis. Subsequently, ch-K5(s-s)R8-An/(Dbait-DOX) micelles were imaged by transmission electron microscopy, and drug loading (DL) and encapsulation efficiency (EE) of DOX were measured by fluorescence spectroscopy. Moreover, the Dbait encapsulation efficiency of ch-K5(s-s)R8-An/(Dbait-DOX) micelles was determined by agarose gel electrophoresis. See Supplementary Material for details.

### 2.4. *In vitro* Dbait and DOX release

Dbait and DOX release was investigated under pH-neutral (pH = 7.4), pH-acidic (pH = 5.5), and reductive acidic (pH = 5.5 + 10 mM dithiothreitol [DTT]) conditions at 37 °C. The release profiles of Dbait and DOX from ch-K5(s-s)R8-An/(Dbait-DOX) micelles (nitrogen/phosphorous [N/P] ratio = 10, polymer:Dbait:DOX ratio = 5.3:1.2:1.0) were measured by agarose gel electrophoresis and spectrophotometry, respectively. See Supplementary Material for details.

### 2.5. *In vitro* micelle uptake and intracellular localization

The cellular uptake and intracellular localization of ch-K5(s-s)R8-An/(Dbait-DOX) were analyzed by imaging YOYO-1 labeled Dbait fluorescence (red), DOX auto fluorescence (green), and 4′,6-diamidino-2-phenylindole (DAPI; cell nuclei, blue) using confocal laser scanning microscopy after incubation with U251 cells for 3 h. The utility of the MMP-2 cleavable linkers in ch-K5(s-s)R8-An micelles was investigated by co-incubation with MMP-2 peptides to simulate the tumor microenvironment. See Supplementary Material for details.

### 2.6. *In vitro* cytotoxicity and anti-proliferation effect

The cytotoxicity and half maximal inhibitory concentration (IC_50_) of the combined treatment with ch-K5(s-s)R8-An/(Dbait-DOX) and RT were assessed with Cell Counting Kit-8 (CCK-8) assays in U251 cells. Colony formation assays were performed to determine the anti-proliferation effect of each treatment. Plating efficiency was used to evaluate the colony formation ability of each group, which was calculated by dividing the colony count by the plated cell count. See Supplementary Material for details.

### 2.7. *In vitro* apoptosis assay

The apoptosis rate of the combined treatment with ch-K5(s-s)R8-An/(Dbait-DOX) and RT was determined by flow cytometry. After treatment, U251 cells were stained by an annexin V-APC/propidium iodide (PI) apoptosis detection kit and analyzed by flow cytometry. Data were processed using FlowJo software. See Supplementary Material for details.

### 2.8. *In vitro* DNA damage and repair

Western blot assays were performed to determine DNA damage and repair in U251 cells after each treatment through the extent of phosphorylation of histone H2A (*γ*-H_2_AX), phospho-P53 (p-P53), and DNA-dependent protein kinase catalytic subunit (DNA-PKcs). See Supplementary Material for details.

### 2.9. *In vivo* brain targeting and biodistribution

Near-infrared fluorescent boron dipyrromethene (BODIPY) was used to investigate the *in vivo* brain targeting ability and biodistribution of the ch-K5(s-s)R8-An micelles. *In situ* U251 tumor-bearing models were constructed by implanting U251 cells into the brain tissue of nude mice. Then, U251 tumor-bearing mice were randomly divided into three groups and were injected with free BODIPY, ch-K5(s-s)R8/BODIPY micelles, or ch-K5(s-s)R8-An/BODIPY through their tail veins. After injection, biodistribution was evaluated by *in vivo* fluorescence imaging and the brain tumor-targeting abilities were assessed by imaging the frozen sections of brain tissues using confocal laser scanning microscopy. See Supplementary Material for details.

### 2.10. *In vivo* anti-glioblastoma efficacy

To evaluate the compound therapeutic effect of ch-K5(s-s)R8-An/(Dbait-DOX) micelles plus RT, *in situ* U251 tumor-bearing mice were randomly allocated to seven groups (*n* = 12/group): control, free DOX, RT, ch-K5(s-s)R8-An/DOX micelles, ch-K5(s-s)R8-An/Dbait micelles + RT, ch-K5(s-s)R8/DOX micelles + RT, and ch-K5(s-s)R8-An/(Dbait-DOX) micelles + RT. Accordingly, micelles, free DOX, vehicle control or physiological saline control were intravenously administered on day 12, 19, and 26 after xenograft implantation (polymer, 10.6 mg/kg; DOX, 2 mg/kg; Dbait, 2.4 mg/kg). Local RT was administered on days 13, 20, and 27 at a cumulative dose of 2 Gy (0.3 Gy/min) into the right brain.

Twenty-four hours after the last RT treatment, intracranial tumors were imaged by magnetic resonance imaging (MRI, Magnetom Aera, Siemens, Munich, Germany) on day 28 after U251 cell implantation. Then, part of the animals were sacrificed (*n* = 6/group) to collect major organs (heart, liver, spleen, lung, kidney, brain), and the remaining animals were kept alive under standard care conditions. The body weight of mice was recorded every two days and survival was monitored to construct Kaplan-Meier curves (*n* = 6/group). The histological damage levels of the brain tumor sections were assessed by hematoxylin and eosin (H&E) staining and *γ*-H_2_AX immunofluorescence staining. Meanwhile, *in vivo* safety of each treatment was evaluated by H&E histological staining of organ sections. See Supplementary Material for details.

### 2.11. Statistical analyses

All data are presented as means ± standard deviation (SD). Statistical analysis was performed using one-way analysis of variance (ANOVA). The difference was considered significant when the *P-*value was less than 0.05.

## 3. Results

### 3.1. Characterization of ch-K5(s-s)R8-an/(Dbait-DOX)

**Figure 1.**
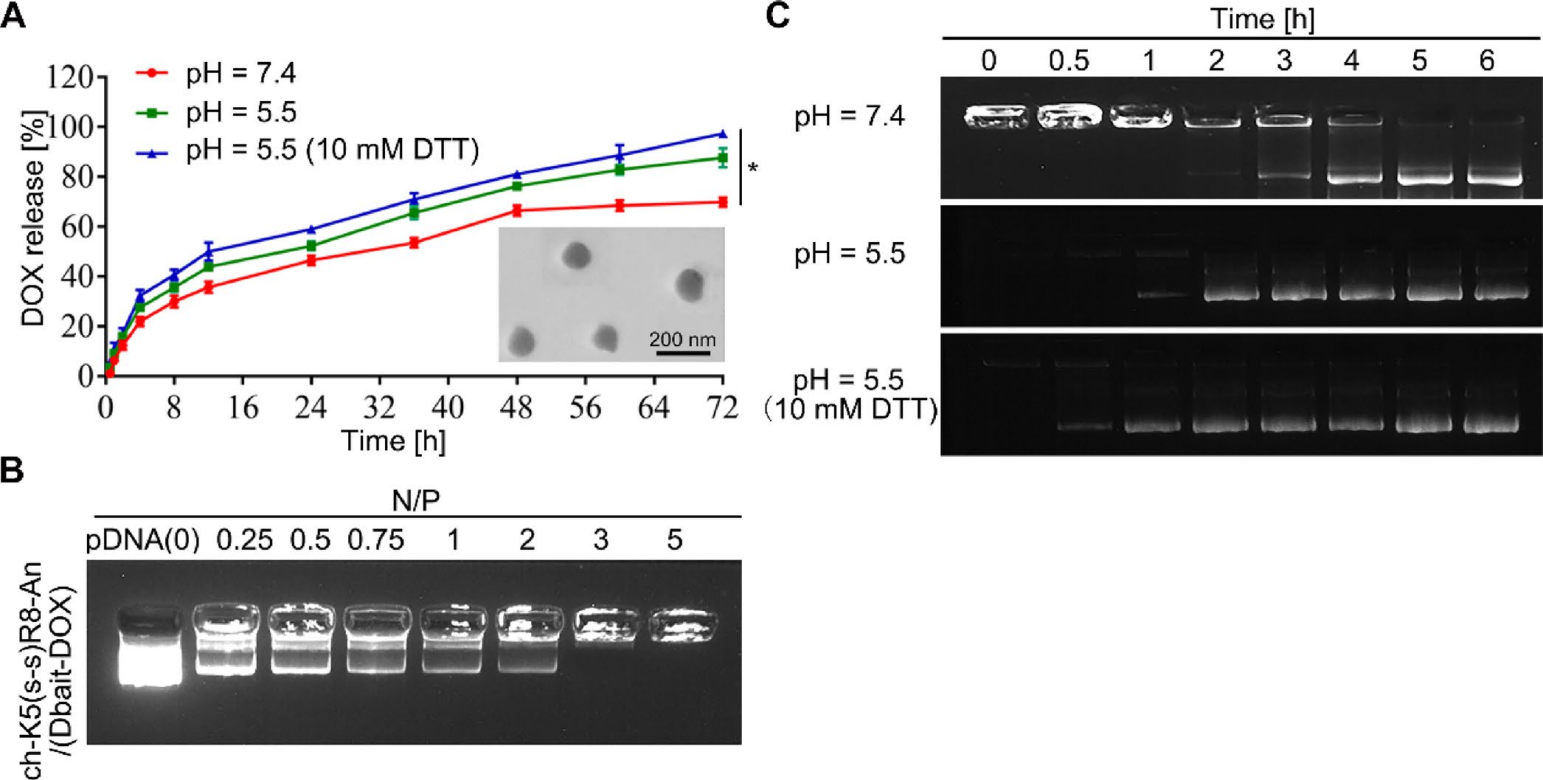
Characterization of ch-K5(s-s)R8-An/Dbait micelles and ch-K5(s-s)R8-An/(Dbait-DOX) micelles. (A) Time-dependent DOX release and TEM image of ch-K5(s-s)R8-An/(Dbait-DOX) micelles. (B) Agarose gel electrophoresis of Dbait binding affinity toward ch-K5(s-s)R8-An/(Dbait-DOX) micelles at an increasing N/P ratio. (C) Time-dependent Dbait release from ch-K5(s-s)R8-An/(Dbait-DOX). (*n* = 3, *P < 0.05).

The following DOX-loaded or/and Dbait-loaded micelles were prepared by membrane dialysis method and the subsequent shell cross-linking reaction: ch-K5(s-s)R8-An/DOX, ch-K5(s-s)R8-An/Dbait, and ch-K5(s-s)R8-An/(Dbait-DOX) (Koo et al., 2012; Shao et al., 2015). The particle size, PDI, and zeta potential of each drug-loaded micelle formulation are presented in Table S1. The ch-K5(s-s)R8-An/(Dbait-DOX) micelles had a spherical morphology with a reasonable diameter (Figure 1A). The zeta potential decreased from 31.6 ± 1.6 mV to 20.2 ± 1.3 mV upon the addition of Dbait to ch-K5(s-s)R8-An/DOX micelles (Table S1), which may be attributed to the anionic character of Dbait (Yao et al., 2016). The DL and EE of DOX in ch-K5(s-s)R8-An/(Dbait-DOX) micelles were 15.8 ± 1.3% and 55.3 ± 2.2%, respectively. The Dbait encapsulation ability of ch-K5(s-s)R8-An/(Dbait-DOX) micelles was determined by agarose gel electrophoresis (Figure 1B). Free Dbait was no longer visible at an N/P ratio ≥ 3, indicating that the micelles (1 mg of polymer) had condensed all of the Dbait (0.6 mg). The incorporation of DOX (0.19 mg) did not affect Dbait condensation by ch-K5(s-s)R8-An/(Dbait-DOX) micelles, suggesting that DOX and Dbait could be co-delivered by these micelles (Yao et al., 2016). In our previous work (Jiao et al., 2019), ch-K5(s-s)R8-An/Dbait micelles showed the most optimal gene transfection and uptake efficiency at an N/P ratio of 10. These results in combination with the finding that ch-K5(s-s)R8-An/(DOX-Dbait) successfully encapsulates Dbait at N/P ratio ≥ 3 led us to employ an N/P ratio of 10 in all subsequent experiments, which accounts for a polymer:Dbait:DOX weight ratio of 5.3:1.2:1.0.

### 3.2. *In vitro* drug release from ch-K5(s-s)R8-an/(Dbait-DOX) micelles

Dbait is a DNA double-strand break mimetic and induces hyperactivation of DNA-dependent protein kinases (DNA-PKs) involved in DNA damage repair. Accordingly, Dbait must enter the nucleus to initiate its radiosensitization effect (Liu et al., 2017). The release profiles of Dbait and DOX from ch-K5(s-s)R8-An/(Dbait-DOX) micelles were investigated under a pH-neutral (pH = 7.4), pH-acidic (pH = 5.5), and reductive acidic (pH = 5.5 + 10 mM DTT) environment at 37 °C in order to simulate physiological conditions in the circulation and the lysosomal compartment in tumor cells (Yao et al., 2016), respectively. The cytosolic glutathione (GSH) concentration in tumor cells is 100-1000 times higher than that in normal cells, which includes glioblastoma (Zheng et al., 2019), thus DTT was used to further mimic the reductive state in cancer cells normally imparted by GSH (Xiong et al., 2018).

At each time point during 4 ∼ 72 h incubation (Figure 1A), DOX release from ch-K5(s-s)R8-An/(Dbait-DOX) micelles under reductive acidic environment (pH = 5.5 + 10 mM DTT) was significantly higher than that under pH-neutral (pH = 7.4, *P* < 0.0001) or non-reductive acidic (pH = 5.5, *P* < 0.05) conditions. In the absence of DTT, DOX release from ch-K5(s-s)R8-An/(Dbait-DOX) micelles was 70 ± 2% and 88 ± 4% at pH = 7.4 and pH = 5.5 after 72 h incubation, respectively, which was augmented to 97 ± 1% by DTT (10 mM, pH = 5.5). As shown in Figure 1C, Dbait release from ch-K5(s-s)R8-An micelles occurred after 3 h of exposure under the simulated normophysiological conditions (pH = 7.4). Notably, the release of Dbait was accelerated to 1 h under acidic conditions (pH = 5.5) and further accelerated to 0.5 h under reductive acidic conditions (pH = 5.5 + 10 mM DTT). These data for DOX and Dbait corroborated the pH-sensitive and redox-sensitive release behaviors of ch-K5(s-s)R8-An micelles.

### 3.3. *In vitro* micelle uptake and intracellular localization

**Figure 2.**
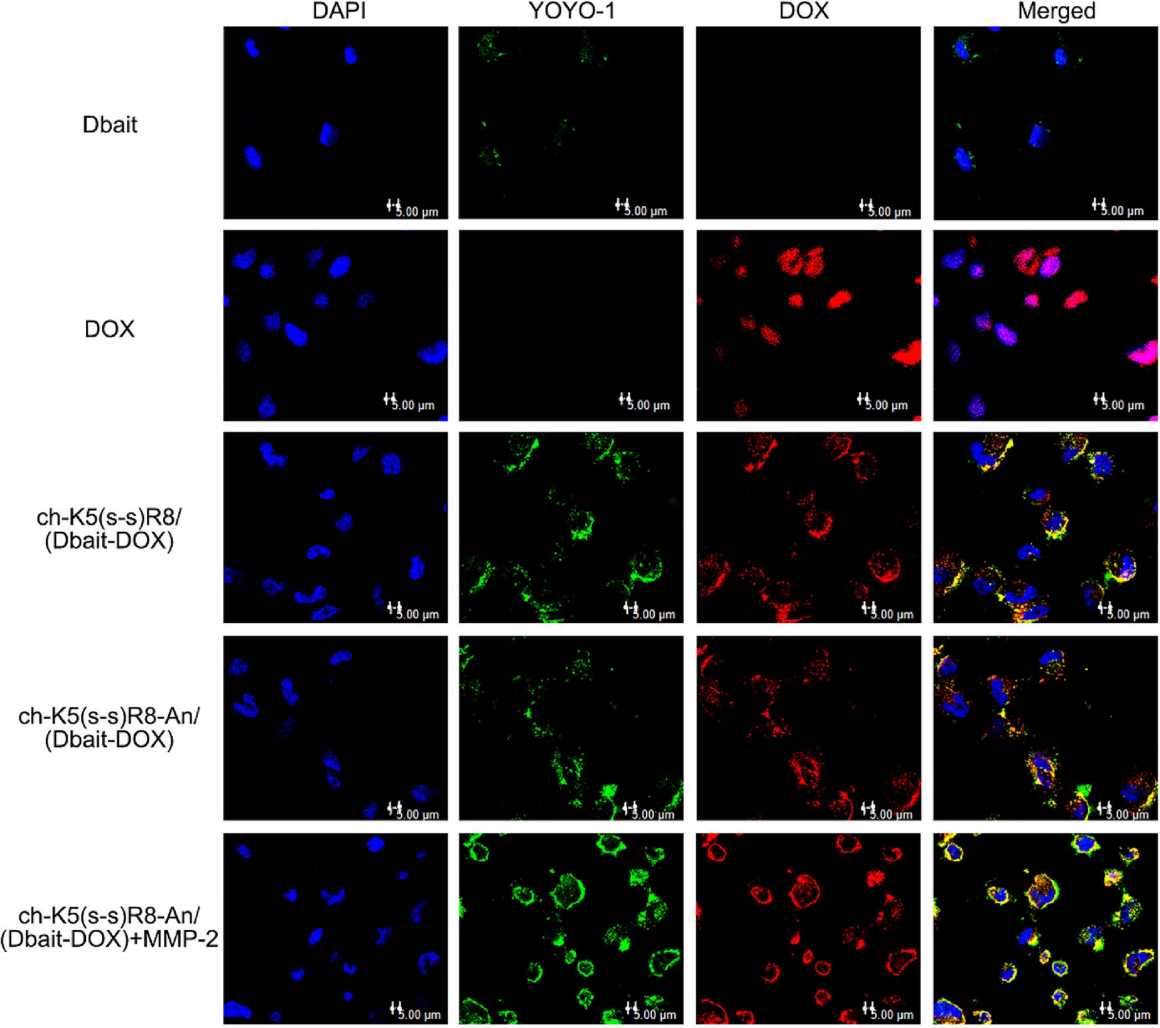
Confocal microscopy images of U251 cells after 3-h incubation with DOX, ch-K5(s-s)R8/(Dbait-DOX) micelles, ch-K5(s-s)R8-An/(Dbait-DOX) micelles, and ch-K5(s-s)R8-An/(Dbait-DOX) micelles + MMP-2. Green fluorescence represents YOYO-1 labeled Dbait, red fluorescence represents DOX, and blue fluorescence represents DAPI-labeled cell nuclei. Scale bar is 5 μm.

The cellular uptake and intracellular localization of ch-K5(s-s)R8-An/(Dbait-DOX) were analyzed by imaging YOYO-1-labeled Dbait (red) and DOX auto fluorescence (green) by confocal laser scanning microscopy after incubation with U251 cells for 3 h (Figure 2). Free Dbait-YOYO-1 was sparsely located in the cytoplasm. Meanwhile, micelle-treated groups exhibited strong Dbait-YOYO-1 fluorescence, which indicated that Dbait significantly benefited from micellar delivery. Free DOX co-localized with DAPI, indicating that the DOX had entered the nuclei during 3-h incubation. In the micelle groups, Dbait-YOYO-1 and DOX were mainly found in the perinuclear regions and partially localized to the nuclei, suggesting that a longer time interval was required to profusely accumulate in the nuclei. Meanwhile, the more abundant cellular fluorescence intensity of ch-K5(s-s)R8/(Dbait-DOX) group and ch-K5(s-s)R8-An/(Dbait-DOX) + MMP-2 group compared to the ch-K5(s-s)R8-An/(Dbait-DOX) without MMP-2 group confirmed that the R8 moiety promoted micelle internalization.

### 3.4. *In vitro* micellar cytotoxicity and anti-proliferation effect of collaborative treatment

**Figure 3.**
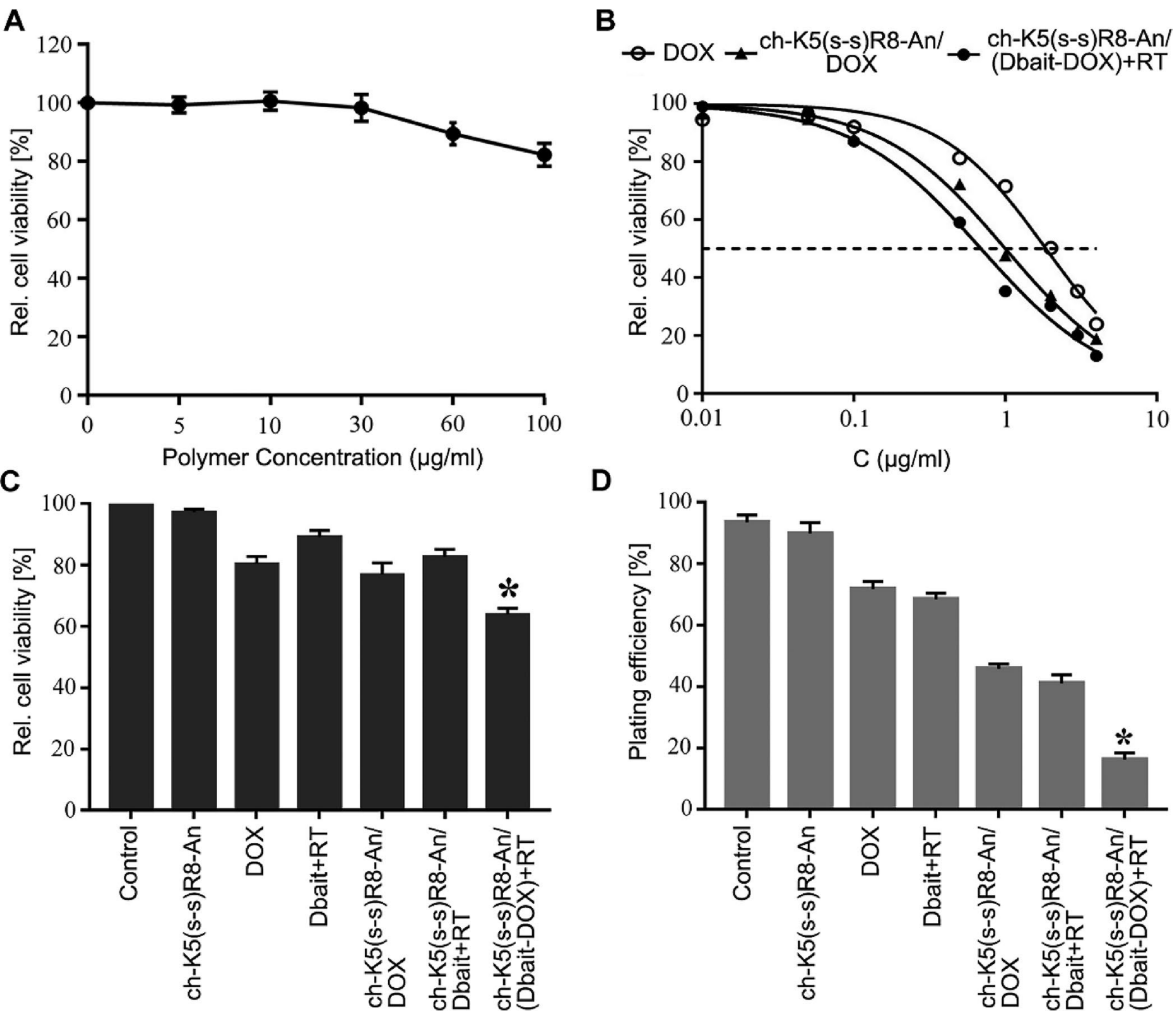
Cytotoxicity and anti-proliferation effects of different micelle formulations in cultured U251 cells. (A) Concentration-dependent cytotoxicity of empty ch-K5(s-s)R8-An micelles. (B) Concentration-dependent toxicity of free DOX, ch-K5(s-s)R8-An/DOX micelles, and ch-K5(s-s)R8-An/(Dbait-DOX) micelles + RT following 48-h incubation. Data were fitted with a linear fit function. (C) Cell viability and (D) colony formation of U251 cells subjected to PBS (control), empty ch-K5(s-s)R8-An micelles, DOX, Dbait + RT, ch-K5(s-s)R8-An/Dbait micelles + RT, ch-K5(s-s)R8-An/DOX micelles, and ch-K5(s-s)R8-An/(Dbait-DOX) micelles + RT. Data in (C,D) were normalized to control (*n* = 3, *P < 0.05).

The toxicity of the different micelle formulations compared to that of free DOX and empty ch-K5(s-s)R8-An micelles was investigated in U251 cells. As shown in Figure 3A, 48-h incubation with empty ch-K5(s-s)R8-An micelles did not produce any toxicity up to a polymer concentration of 30 μg/mL. Therefore, the toxicity observed in cells treated with DOX, ch-K5(s-s)R8-An/DOX micelles, and ch-K5(s-s)R8-An/(Dbait-DOX) micelles in combination with RT were attributed to the DOX and Dbait/RT regimens and not the polymer carrier (Figure 3B). The ch-K5(s-s)R8-An/(Dbait-DOX) micelles plus RT exhibited the lowest IC_50_ (0.692 μg/mL), which was approximately 2.7- and 1.5-fold lower than that of free DOX and ch-K5(s-s)R8-An/DOX micelle-delivered DOX, respectively (Figure 3B).

The synergistic therapeutic efficacy of ch-K5(s-s)R8-An/(Dbait-DOX) micelles was further investigated (Figure 3C). In agreement with Figure 3A, empty K5(s-s)R8-An micelles (2.6 µg/mL polymer concentration) exerted no toxicity, as shown in Figure 3C. Following 48-h treatment with Dbait (0.6 µg/mL) and DOX (0.5 µg/mL), cell viability was reduced in the following order relative to control: Dbait + RT (89.2 ± 2.1%), ch-K5(s-s)R8-An/Dbait micelles + RT (82.7 ± 2.4%), DOX (80.5 ± 2.4%), and ch-K5(s-s)R8-An/DOX micelles (76.9 ± 3.8%). Treatment with ch-K5(s-s)R8-An/(Dbait-DOX) + RT reduced cell viability to 64.0 ± 2.1%, corresponding to 1.6-fold (*P* < 0.001) and 2.1-fold (*P* < 0.0001) greater lethality than ch-K5(s-s)R8-An/DOX micelles and ch-K5(s-s)R8-An/Dbait micelles + RT, respectively.

Colony formation is standardly employed to assess the long-term cell proliferation capacity (Ding et al., 2017). As shown in Figure 3D, the plating efficiency of control U251 cells was most prominent (93.5 ± 2.4%), followed by U251 cells treated with Dbait + RT (89.8 ± 3.6%), empty ch-K5(s-s)R8-An micelles (71.8 ± 2.4%), DOX (68.5 ± 2.0%), ch-K5(s-s)R8-An/DOX micelles (45.8 ± 1.5%), ch-K5(s-s)R8-An/Dbait micelles + RT (41.0 ± 2.9%), and ch-K5(s-s)R8-An/(Dbait-DOX) micelles + RT (16.3 ± 2.1%). Taken together, these results confirmed that the micelles containing DOX and Dbait combined with RT acted synergistically on cultured glioblastoma cells in terms of increasing cytotoxicity and decreasing proliferation.

### 3.5. *In vitro* apoptosis-inducing effect of combined treatment

**Figure 4.**
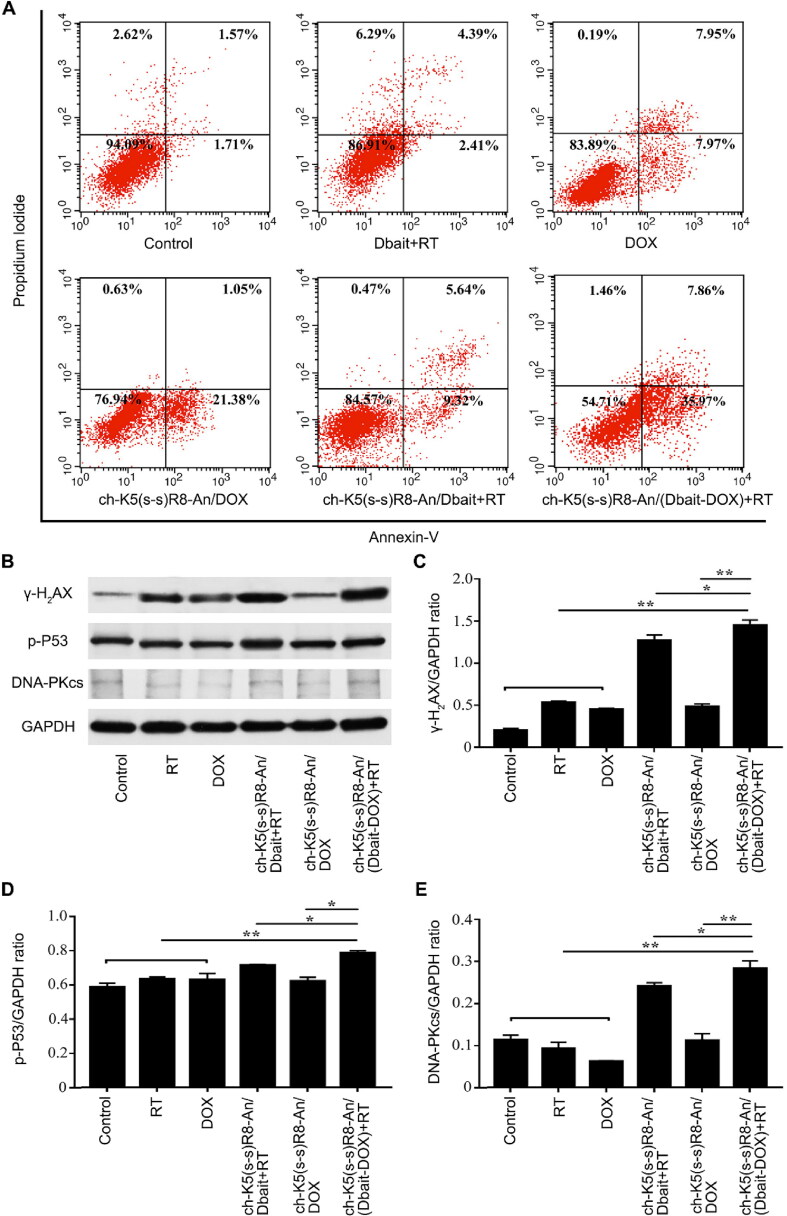
*In vitro* apoptosis and DNA damage response of different micelle formulations in cultured U251 cells. (A) Flow cytometric analysis of apoptosis in U251 cells induced by ch-K5(s-s)R8-An micelles, DOX, Dbait micelles + RT, ch-K5(s-s)R8-An/Dbait micelles + RT, ch-K5(s-s)R8-An/DOX micelles, and ch-K5(s-s)R8-An/(Dbait-DOX) micelles + RT. (B-E) Protein samples of U251 cells were collected 48 h after RT for Western blot analysis. The protein band intensities of the DNA double-strand break marker *γ*-H_2_AX (C), DNA damage repair proteins phospho-p53 (D) and DNA-PKcs (E) were quantified by densitometric analysis and normalized to the respective GAPDH band intensity (*n* = 3, *P < 0.05, **P < 0.01).

DOX is known to mainly induce apoptosis (Kalyanaraman et al., 2002). The manifestation of apoptosis was more pronounced when DOX was delivered *via* nanocarriers (Zeng et al., 2021). To determine the extent of apoptosis induced by different formulations, U251 cells were stained with annexin-V/PI and assayed by flow cytometry. The rate of apoptosis proceeded in the following order: control cells (3.4 ± 0.3%), Dbait + RT (6.8 ± 0.7%), DOX (15.9 ± 0.7%), ch-K5(s-s)R8-An/DOX micelles (22.4 ± 0.9%), and ch-K5(s-s)R8-An/Dbait micelles + RT (15.0 ± 0.6%) (Figure 4A). Moreover, ch-K5(s-s)R8-An/(Dbait-DOX) micelles + RT treatment caused apoptosis in 43.8 ± 1.1% of cells, corresponding to a 1.9-fold (*P* < 0.001) increase in the rate of apoptosis compared to cells in the ch-K5(s-s)R8-An/DOX treatment group and a 2.9-fold (*P* < 0.0001) increase relative to cells in the ch-K5(s-s)R8-An/Dbait + RT treatment group. The findings were in agreement with the CCK-8 assay, which indicated that micellar delivery potentiates DOX chemotherapeutic efficacy *in vitro* and reiterated the synergistic effect of micellar DOX and Dbait in combination with RT in cultured glioblastoma cells.

### 3.6. *In vitro* DNA damage response to combined treatment

RT and DOX are known to produce DNA double-strand breaks (Biau et al., 2019). Dbait promotes phosphorylation of nuclear DNA-PK targets and prevents DNA damage repair proteins from detecting actual chromosome double-strand breaks (Giovannini et al., 2014). Nuclear *γ*-H_2_AX serves as an indicator of DNA double-strand breaks and will accumulate when the DNA double-strand breaks are not repaired (Kavanagh et al., 2013; Yao et al., 2016). γ-H2AX levels were therefore analyzed by Western blot. As shown in Figure 4B and C, U251 cells treated with ch-K5(s-s)R8-An/(Dbait-DOX) + RT expressed significantly higher levels of *γ*-H_2_AX compared to those in the ch-K5(s-s)R8-An/Dbait + RT and ch-K5(s-s)R8-An/DOX treatment group. Levels of DNA repair proteins phospho-P53 (p-P53, Figure 4D) and DNA-PKcs (Figure 4E) were consistent with the extent of DNA double-strand breaks (*γ*-H_2_AX, Figure 4C) after ch-K5(s-s)R8-An/(Dbait-DOX) + RT treatment. These results suggested that ch-K5(s-s)R8-An/(Dbait-DOX) + RT synergistically improve RT efficacy by raising the DNA damage level and duration in U251 cells.

### 3.7. *In vivo* brain targeting and biodistribution of ch-K5(s-s)R8-An micelles

**Figure 5.**
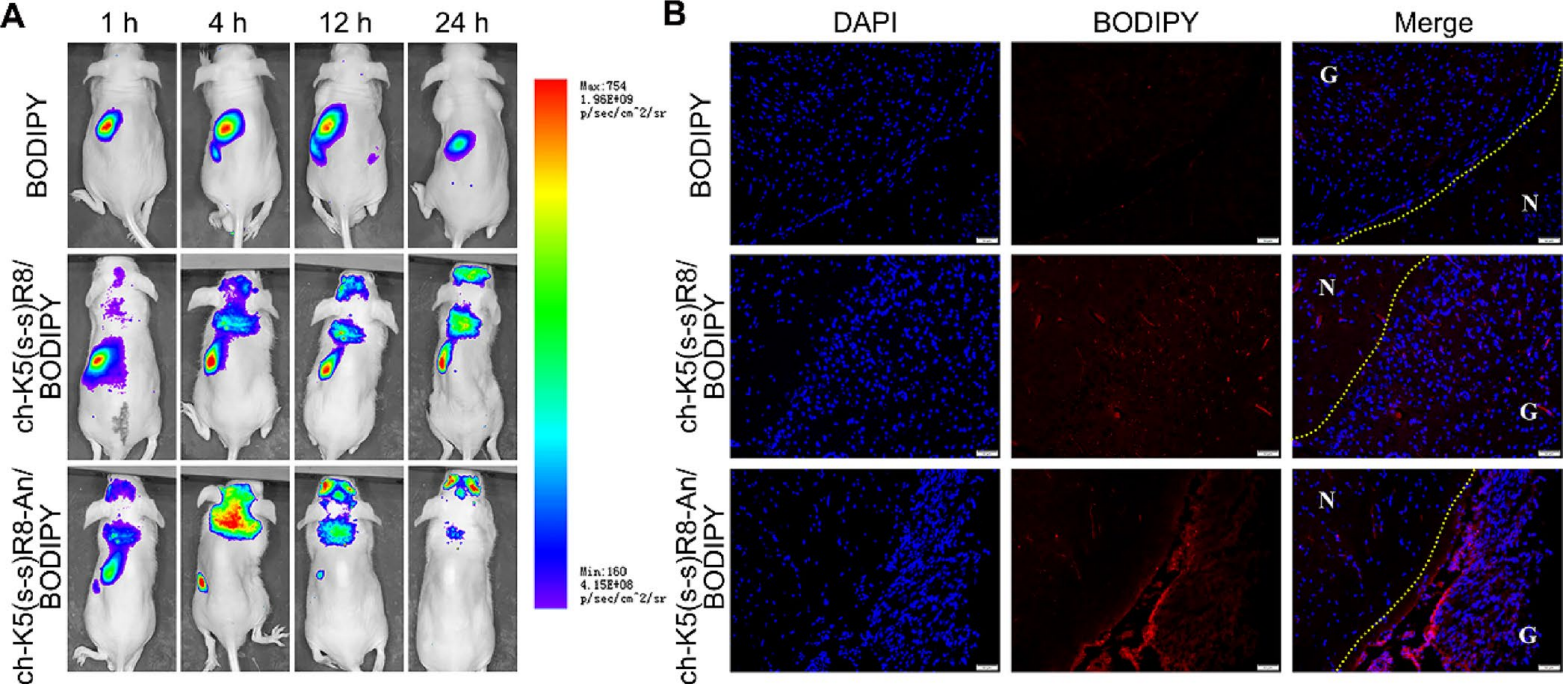
Intravital and postmortem organ imaging of BODIPY-labeled micelles in U251 tumor-bearing nude mice (A) Real-time *in vivo* fluorescence imaging of U251 tumor-bearing nude mice after intravenous injection of BODIPY or BODIPY-labeled micelles at 1, 4, 12, and 24 h. (B) Fluorescence microscopy images showed the distribution of BODIPY (red) in glioblastoma xenografts 4 h after intravenous injection of BODIPY or BODIPY-labeled micelles. Nuclei were stained with DAPI (blue). Scale bar is 50 µm. G: glioblastoma; N: normal brain tissue.

Investigation of *in vivo* biodistribution is essential for the evaluation of the safety and potential efficacy of drug delivery systems (Feng et al., 2014). As illustrated in Figure 5A, no free BODIPY fluorescence (negative control) was detected in the cranial region of glioblastoma-bearing mice during 24 h of circulation, which was confirmed in the postmortem brain (Figure 5B). In contrast, BODIPY labeled ch-K5(s-s)R8 and ch-K5(s-s)R8-An micelles were observed in the brain area at 4 h after injection and their brain accumulation gradually increased during the subsequent 20 h (Figure 5A), which lead to high BODIPY fluorescence in the excised brains of mice treated with ch-K5(s-s)R8-An/BODIPY micelles. Confocal laser scanning microscopy of histological sections of excised brains revealed that ch-K5(s-s)R8-An/BODIPY micelles and ch-K5(s-s)R8/BODIPY micelles had accumulated in the tumor site 4 h after intravenous injection (Figure 5C). Strong BODIPY fluorescence was observed in the tumor tissues with ch-K5(s-s)R8-An/BODIPY micelles, which underscored the brain-targeting utility of angiopep-2 and the *in situ* glioblastoma-targeting ability of ch-K5(s-s)R8-An micelles.

### 3.8. *In vivo* anti-glioblastoma efficacy of combined treatment

*In vivo* anti-tumor effect of different formulations and the synergistic effect of ch-K5(s-s)R8-An/(Dbait-DOX) micelles combined with RT were further investigated from multiple angles in U251 tumor-bearing nude mice.

**Figure 6.**
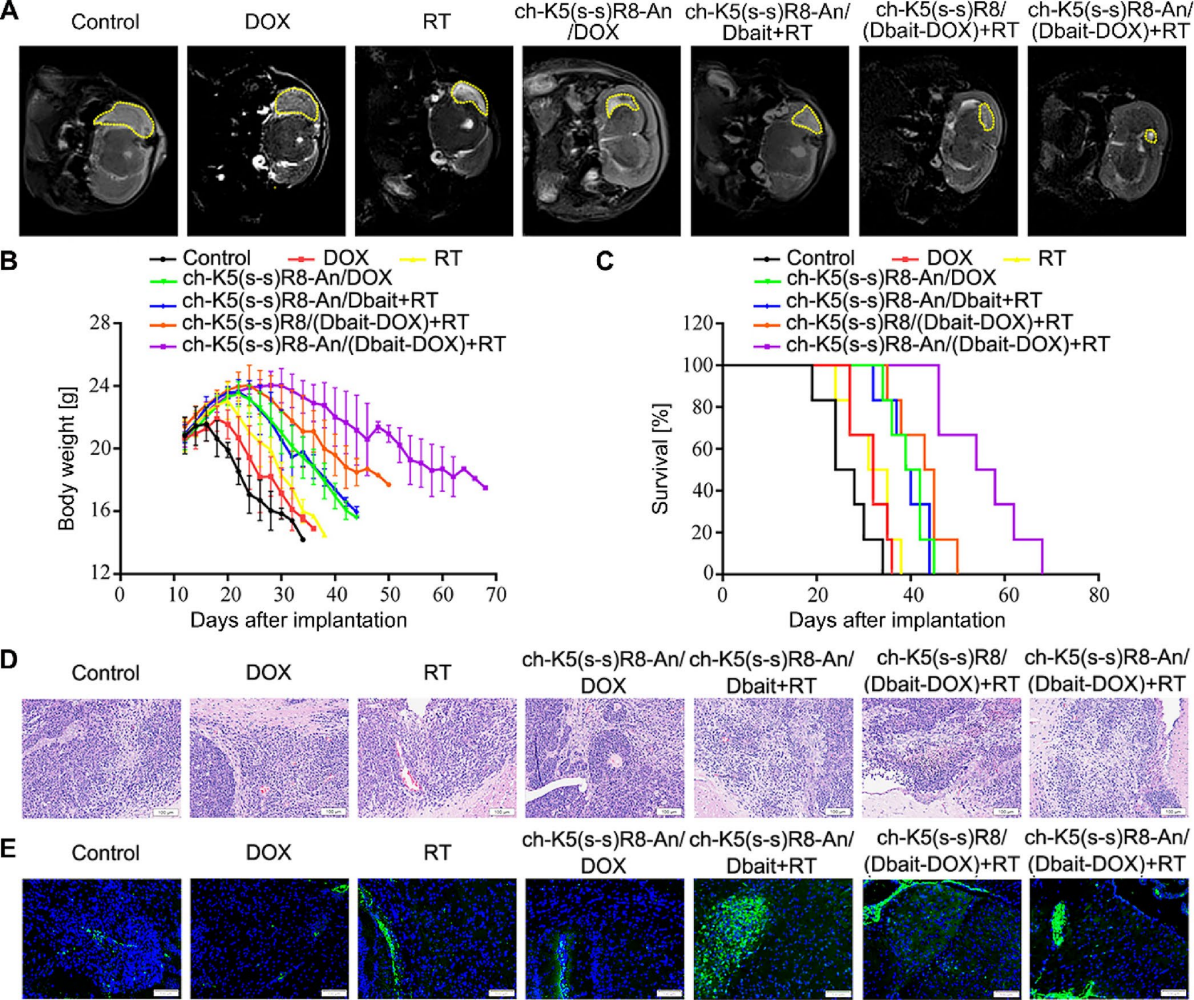
*In vivo* micelle-mediated chemo-radiotherapy sensitization in orthotopic U251 tumor-bearing nude mice. (A) Representative MRI images of orthotopic U251 glioblastoma on day 28 after xenograft implantation. (B) Body weight and (C) Kaplan-Meier survival curves of orthotopic U251 tumor-bearing nude mice. (D) Histological characteristics and (E) *γ*-H_2_AX immunofluorescence (green) of orthotopic U251 tumor sections. Cell nuclei were stained with DAPI (blue). Scale bar is 100 µm. (*n* = 6, *P < 0.05, **P < 0.01).

First, intracranial tumors were imaged and visualized by brain MRI. As shown in Figure 6A, tumors in control mice that had received physiological saline were the largest, while mice treated with DOX alone exerted only a modest inhibitory effect on intracranial tumor growth. In contrast, therapeutic efficacy was observed in the following order: RT < ch-K5(s-s)R8-An/DOX micelles ≈ ch-K5(s-s)R8-An/Dbait micelles + RT < ch-K5(s-s)R8/(Dbait-DOX) micelles + RT < ch-K5(s-s)R8-An/(Dbait-DOX) micelles + RT. The tumor inhibitory effect was most pronounced in the ch-K5(s-s)R8-An/(Dbait-DOX) micelles + RT group compared to the other groups, highlighting the sensitizing effect of DOX and Dbait combined with RT, which further proved that angiopep-2-modified micelles could enhance the antitumor efficacy due to the brain-targeting attributes *in vivo*.

Second, therapeutic efficacy was measured by monitoring body weight (Figure 6B) and Kaplan-Meier survival analysis (Figure 6C, Table S2), which are negative indicators that are affected by the growth of orthotopic glioblastoma (Lee et al., 2012; Shan et al., 2016). The body weight of tumor-bearing mice in the control group began to decrease rapidly 2 weeks after tumor cell inoculation. Mice that had been treated with ch-K5(s-s)R8-An/(Dbait-DOX) micelles + RT experienced the longest post-inoculation weight gain and the least weight deterioration. When compared with the saline group, the median survival times of mice treated with free DOX, RT, ch-K5(s-s)R8-An/Dbait + RT, ch-K5(s-s)R8-An/DOX, and ch-K5(s-s)R8-An/(Dbait-DOX) + RT were increased by 23.1%, 26.92%, 51.9%, 55.8% and 69.2%, respectively (Table S2). In contrast, ch-K5(s-s)R8/(Dbait-DOX) + RT combination treatment was the most effective treatment, with a 115.4% median survival extension compared to the saline treatment by increasing the median survival time from 26 days to 56 days. Those results indicated that the ch-K5(s-s)R8-An/(Dbait-DOX) co-delivery system can synergistically act with RT by suppressing U251 solid tumors and increasing the survival of tumor-bearing mice *in vivo*.

Third, we assessed histological tissue damage (Figure 6D) and *γ*-H_2_AX immunofluorescence (Figure 6E) in tumor biopsies after the completion of therapeutic regimens. Compared to other groups, mice in the ch-K5(s-s)R8-An/(Dbait-DOX) micelles + RT group exhibited a higher extent of nuclear pyknosis and liquefactive necrosis, with an increased γ-H2AX level in orthotopic tumor sections. These results confirmed the *in vivo* synergistic cytotoxicity and DNA damaging effect of the chemo-radiotherapy where ch-K5(s-s)R8-An/(Dbait-DOX) micelles were used as the radiosensitizer delivery system.

**Figure 7.**
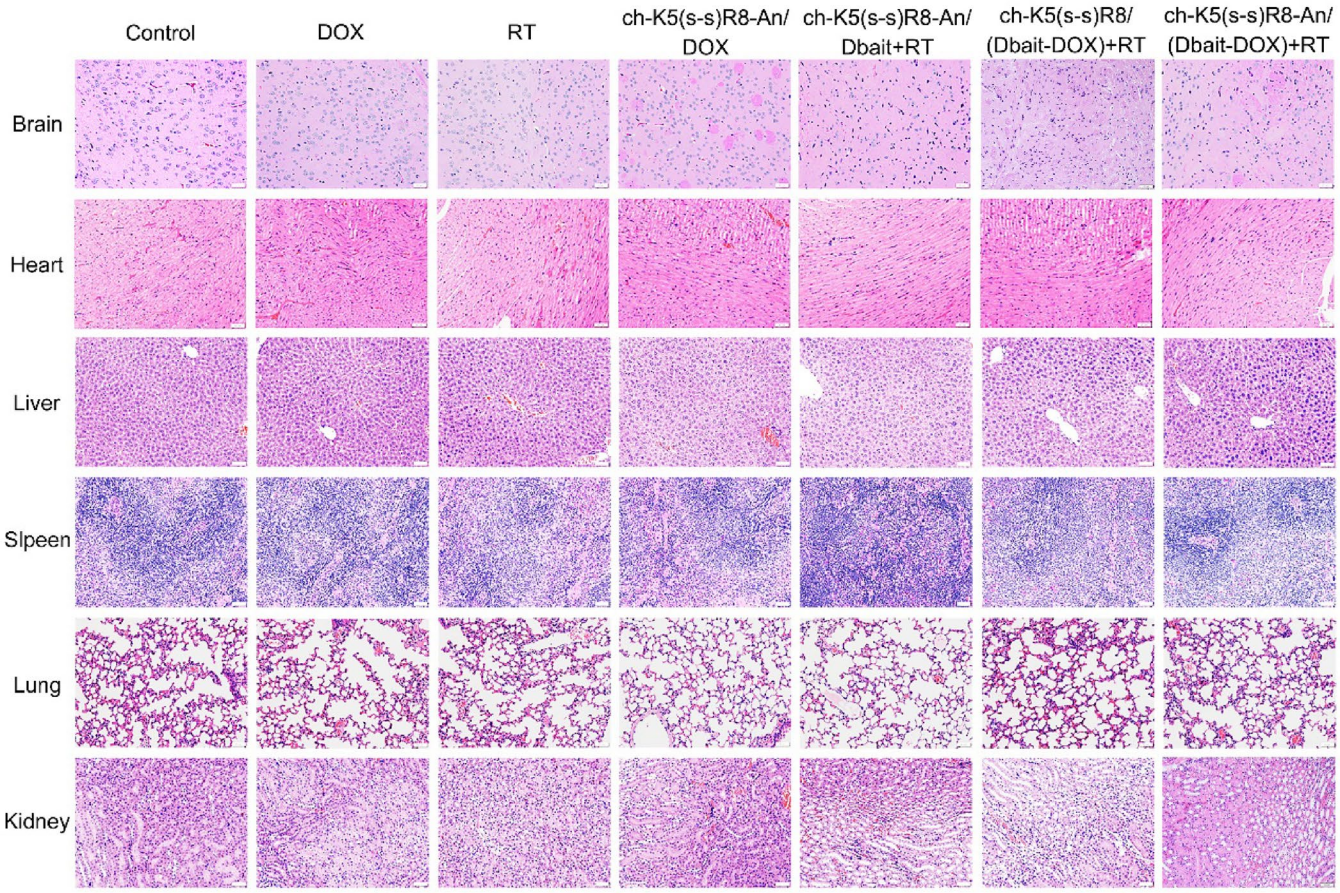
Representative histological micrographs of main organs following the administration of saline, free DOX, RT, ch-K5(s-s)R8-An/DOX, ch-K5(s-s)R8-An/Dbait + RT, ch-K5(s-s)R8/(Dbait-DOX) + RT, ch-K5(s-s)R8-An/(Dbait-DOX) + RT. Scale bar is 100 µm.

In addition, histological analysis was performed to examine the *in vivo* safety of systemic administration of ch-K5(s-s)R8-An/(Dbait-DOX) micelles and RT (Figure 7). In agreement with the reported cardiotoxicity of DOX (Zou et al., 2018), mice in the free DOX group showed cardiac muscle damage in the form of distorted, swollen, denatured myocardial cells and disorganized, fractured myocardial fibers that appeared in a wavy pattern. However, no obvious cardiotoxicity was observed in any of the micelle-treated or RT-treated mice compared to the control group. Also, none of the micellar formulations imparted *in vivo* toxicity to the brain, liver, spleen, lungs, and kidneys, indicating the *in vivo* safety of the combination treatment with systemically administered micelles and the locally administered radiation in mice.

## 4. Discussion

In this study, we have developed a novel brain-targeting and tumor microenvironment-responsive micelle formulation (ch-K5(s-s)R8-An) for the co-delivery of radiosensitizer Dbait and the chemotherapeutic drug DOX into malignant glioblastoma tumors. This Dbait and DOX co-delivery system also showed high drug loading capacity and good drug release behavior *in vitro*. The mean diameter of these micelles favors a long circulation time and renders the micelles suitable for systemic administration and tumor targeting *via* an enhanced permeability and retention effect (Acharya & Sahoo, 2011; Li et al., 2022). Also, the positive zeta potential of ch-K5(s-s)R8-An/(Dbait-DOX) is conducive to electrostatic interactions between the drug delivery system and the negatively charged cancer cell membrane, which may facilitate greater uptake efficiency and accumulation in tumor tissue (Perrault et al., 2009; Kuo et al., 2018). In addition, its environment-dependent release kinetics are favorable for the release of micelle-delivered Dbait and DOX in tumor tissue (You et al., 2021). Based on the results, it is expected that the micelle particles will remain relatively stable and retain Dbait and DOX in plasma. Once delivered into tumor cells, Dbait and DOX are released into the cytosol under the lysosomal acidic environment and cytoplasmic reductive environment (Wang et al., 2015).

In our previous observations (Jiao et al., 2019), ch-K5(s-s)R8-An micelles exit lysosomes by raising the lysosomal pH, leading to osmotic swelling and bursting of lysosomes. Therefore, it is anticipated that the cytoplasmic release and nuclear transport of micellar Dbait and DOX occur after the lysosome escape and disulfide bond dissociation in the ch-K5(s-s)R8-An/(Dbait-DOX) micelles. In this study, ch-K5(s-s)R8-An/(Dbait-DOX) micelles promoted the uptake of Dbait and DOX through a different mechanism than that of free DOX (Lee et al., 2008; Gong et al., 2017), which is consistent with an earlier report that free DOX passively and rapidly diffuses into nuclei after 2-h incubation whilst nanoparticulate DOX is still retained in the cytoplasm (Prabaharan et al., 2009). Furthermore, the ch-K5(s-s)R8-An micelles showed rapid accumulation in the *in situ* glioblastoma xenografts after intravenous administration, with the most abundant accumulation occurring in the brain region of glioblastoma-bearing mice. This positive brain-targeting property of ch-K5(s-s)R8-An micelles was consistent with our previous report (Jiao et al., 2019), indicating that the angiopep-2 modification effectively enhances the tumor accumulation of ch-K5(s-s)R8-An micelles due to its brain-targeting characteristics.

In addition, the co-delivery system combined with RT treatment exhibited a synergistic anti-glioblastoma effect in U251 cells and *in situ* U251 tumor xenografts. Even though the DNA damage level (Figure 4C) was just slightly greater than that induced by ch-K5(s-s)R8-An/Dbait + RT treatment, ch-K5(s-s)R8-An/(Dbait-DOX) + RT treatment still contributed to a more marked cell death rate (Figure 3C) and apoptotic phenotype (Figure 4A) at 48 h post-RT. This notable phenomenon suggested that the addition of DOX to Dbait + RT therapy expedites and/or augments the extent of cell death. In line with *in vitro* results, ch-K5(s-s)R8-An/Dbait + RT treatment yielded the smallest tumor size, strongest tumor tissue injury, and highest DNA damage in orthotopic U251 tumors, which increased the median survival time of U251 tumor-bearing mice from 26 days to 56 days. These results highlighted the complementary therapeutic potency of the combined ch-K5(s-s)R8-An/(Dbait-DOX) plus RT treatment.

In summary, we developed a novel brain-targeting and tumor microenvironment-responsive micelle formulation (ch-K5(s-s)R8-An) for the co-delivery of radiosensitizer Dbait and the chemotherapeutic drug DOX into malignant glioblastoma. This ch-K5(s-s)R8-An co-delivery system showed good glioblastoma targeting specificity and reduced DOX-mediated cardiotoxicity. The combined chemo-radiotherapy of ch-K5(s-s)R8-An/(Dbait-DOX) plus RT exhibited the most remarkable *in situ* tumor inhibition outcome with smaller tumor size, longer median survival time, and higher DNA damage rate than any other separate treatment. Therefore, this dual-targeting and microenvironment-responsive micellar system shows promise as an advanced co-delivery system for Dbait and hydrophobic drugs such as DOX for the chemo-radiotherapy of malignant glioblastoma.

## Supplementary Material

Supplemental Material

## Supplementary material

Supplementary Material includes the following sections: Materials and methods; Table S1: Particle characterization of different micelle formulations; and Table S2: Median survival time of tumor-bearing mice after different treatments. (DOCX)
