# Supplementary material for: A tumor microenvironment-responsive micelle co-delivered radiosensitizer Dbait and doxorubicin for the collaborative chemo-radiotherapy of glioblastoma
Source: Drug Deliv. 2022 Aug 16;29(1):2658–70. doi: 10.1080/10717544.2022.2108937 (PMC9387324; doi:10.1080/10717544.2022.2108937)
Supplement: Supplemental Material [file IDRD_A_2108937_SM6363.docx]

A tumor microenvironment-responsive micelle co-delivered radiosensitizer Dbait and doxorubicin for the collaborative chemo-radiotherapy of glioblastoma

Shuyue Zhang^1‡^, Xiuxiu Jiao^1,2‡^, Michal Heger^3^, Shen Gao^4^, Mei He^1^, Nan Xu^1^, Jigang Zhang^1^, Mingjian Zhang^1^, Yuan Yu^4^*, Baoyue Ding^3^*, Xueying Ding^1^*

1 Shanghai General Hospital, Shanghai Jiao Tong University School of Medicine, Shanghai, China

2 Department of Pharmacy, Shanghai 9th People's Hospital, Shanghai Jiao Tong University School of Medicine, Shanghai, China

3 Laboratory of Oncological Photodynamic Therapy and Targeted Drug Research, College of Medicine, Jiaxing University, Jiaxing, China

4 Department of Pharmaceutical Science, School of Pharmacy, Naval Medical University, Shanghai, China

*Corresponding Authors. Tel.: +86-21-36126307; fax: +86-21-56095970

E-mail addresses: dingxueying@126.com (Xueying Ding), lena_310@163.com (Baoyue Ding), pharmyuu@163.com (Yuan Yu).

^‡^These authors contributed equally to this work.

Table of contents

[Materials and methods 3](#_Toc108796559)

[1. Materials 3](#_Toc108796560)

[2. Cell lines and animals 3](#_Toc108796561)

[3. Micelle preparation and characterization 4](#_Toc108796562)

[4. *In vitro* Dbait and DOX release 5](#_Toc108796563)

[5. *In vitro* micelle uptake and intracellular localization 6](#_Toc108796564)

[6. *In vitro* cytotoxicity and anti-proliferation effect 7](#_Toc108796565)

[7. *In vitro* apoptosis assay 8](#_Toc108796566)

[8. *In vitro* DNA damage and repair 8](#_Toc108796567)

[9. *In vivo* brain targeting and biodistribution 9](#_Toc108796568)

[10. *In vivo* anti-tumor efficacy 10](#_Toc108796569)

[11. Statistical analyses 12](#_Toc108796570)

[Table S1. Particle characterization of different micelle formulations. 13](#_Toc108796571)

[Table S2. Median survival time of tumor-bearing mice after different treatments 13](#_Toc108796572)

[References 14](#_Toc108796573)

**Materials and methods**

1. **Materials**

The cholesterol-KKKKKRRRRRRRR (ch-K5R8) and cholesterol-KKKKKRRRRRRRR-Angiopep-2 (ch-K5R8-An) peptides were designed by our group and then synthesized by Ontores Biotechnologies (Shanghai, China) at > 95% purity. Other materials used in this study were: DOX hydrochloride (99%) (Hisun Pharmaceutical, Zhejiang, China; hydrochloric acid was removed before use); Dbait (Wuhan GeneCreate Biological Engineering, Wuhan, China); 4′,6-diamidino-2-phenylindole (DAPI, Cayman Chemical, Ann Arbor, MI, USA); BODIPY (4,4-difluoro-5,7-dimethyl-4-bora-3a, 4a-diaza-s-indacene-3-propionic acid, sulfosuccinimidyl ester, sodium salt) and YOYO-1 iodide (Thermo Fisher Scientific, Waltham, MA, USA); Cell Counting Kit-8 (CCK-8, Dojindo Molecular Technologies, Kumamoto, Japan); Annexin V-APC Apoptosis Analysis Kit (eBioscience, San Diego, CA, USA); dimethyl sulfoxide (DMSO) and pyrene (99%) (Sangon Biotech, Shanghai, China); 3,3'-dithiobis(sulfosuccinimidylpropionate) (DTSSP, J&K Chemical, Beijing, China); recombinant human MMP-2 proenzyme (Peprotech, Rocky Hill, NJ, USA); Angiopep-2 (TFFYGGSRGKRNNFKTEEY, Ontores Biotechnologies, Shanghai, China).

1. **Cell lines and animals**

The human malignant glioblastoma cell line U251 was obtained from the Type Culture Collection of the Chinese Academy of Sciences (Shanghai, China). Cells were seeded in a 75 cm^2^ culture flask and grown in Dulbecco’s modified Eagle’s medium (DMEM) (Gibco, USA) supplemented with 10% fetal bovine serum (FBS) (Gibco, USA) and 100 U/mL penicillin-streptomycin (Gibco, USA) at 37 °C in a humidified atmosphere containing 5% CO_2_ (standard culture conditions) until 80% conﬂuence.

Male Balb/c nude mice (18-22 g, N = 100) were purchased from the Department of Experimental Animals of the Second Military Medical University (Shanghai, China). The mice were housed in temperature- and humidity-controlled individually ventilated cages with free access to gamma-sterilized food and sterilized water. All animal experiments were performed in compliance with the *National Institute of Health Guidelines for the Care and Use of Laboratory Animals* (8^th^ edition) and institutional guidelines following approval of the institutional review board of Shanghai Jiao Tong University.

1. **Micelle preparation and characterization**

The ch-K5(s-s)R8 micelles, ch-K5(s-s)R8-An micelles, ch-K5(s-s)R8-An/DOX micelles, ch-K5(s-s)R8-An/Dbait micelles, and ch-K5(s-s)R8-An/(Dbait-DOX) micelles were prepared by membrane dialysis method and a subsequent shell cross-linking reaction ^1,2^. Ch-K5(s-s)R8 and ch-K5(s-s)R8-An polymers (5 mg) were dissolved in 5 mL of DMSO, with 50% (molar ratio of total copolymers) ch-K5R8-An added to the copolymer-DMSO solution. DOX·HCl (2 mg) was dissolved in 3 mL of DMSO containing 3× molar excess of triethylamine. The DOX solution was injected into the copolymer-DMSO solution. Then, the mixed solution was dialyzed against 1 L of deionized water for 48 h using a dialysis membrane with a molecular weight cut-off (MWCO) of 3,000 Da (Pierce, Rockford, IL, USA). Next, DTSSP was used as a disulfide-containing cross-linking agent and added to the solution at a [DTSSP]:[Lys] molar ratio = 1:1. The reaction was maintained for 4 h at pH = 8.0, after which the solution was dialyzed for 4 h to remove residual DTSSP. Finally, a microfiltration membrane (0.45 μm pore size) was used to remove insoluble DOX in the micellar dispersion. Empty micelles (ch-K5(s-s)R8-An) were prepared in the same manner as described for ch-K5(s-s)R8-An/DOX, but without DOX. Ch-K5(s-s)R8-An/(Dbait-DOX) and ch-K5(s-s)R8-An/Dbait were prepared by adding Dbait into ch-K5(s-s)R8-An/DOX and ch-K5(s-s)R8-An at N/P ratio = 10, followed by vortexing for 30 s and incubation for 30 min at room temperature.

Particle size and zeta potential of ch-K5(s-s)R8-An/DOX and ch-K5(s-s)R8-An/(Dbait-DOX) (N/P ratio = 10) were measured in PBS (pH = 7.4) by dynamic light scattering (Zetasizer Nano ZS90, Malvern Instruments, Malvern, UK). The morphology of the ch-K5(s-s)R8-An/(Dbait-DOX) was imaged by transmission electron microscope (TEM, Hitachi, Tokyo, Japan) at an acceleration voltage of 75 kV (Hitachi, Tokyo, Japan). DL and EE of ch-K5(s-s)R8-An/(Dbait-DOX) were measured by fluorescence spectroscopy. The ch-K5(s-s)R8-An/(Dbait-DOX) micelles (N/P ratio = 10, polymer:Dbait ratio = 5.3:1.2) were diluted 10-fold in DMSO and ultrasonicated for 20 min to disrupt the micelles and dissolve the DOX. The absorption of the mixture was measured spectrophotometrically (UV-2600, Shimadzu, Kyoto, Japan) at 480 nm. A DOX standard curve (0.5-10 μg/mL DMSO) and the corresponding linear fit function were used to determine the concentration of DOX. DL and EE were calculated using the following equations:

$$DL=\frac{DOX loaded into micelles}{total weight of micelles}\times100\%$$

$$EE=\frac{DOX loaded into micelles}{theoretical amount of DOX loaded into micelles}\times100\%$$

Moreover, the Dbait encapsulation ability of ch-K5(s-s)R8-An/(Dbait-DOX) micelles was determined through agarose gel electrophoresis method. The ch-K5(s-s)R8-An/(Dbait-DOX) micelles were prepared at different N/P ratios (N/P ratio = 0~5). After 30 min incubation, the samples were separated on a 1.0% agarose gel (100 V for 40 min) and stained with the Dbait-specific ﬂuorescent dye Gelred (Biotium, Hayward, CA, USA) in 1 × Tris-acetate EDTA buffer (pH = 8.0) for 40 min at 4 °C. The gels were visualized using a UV transilluminator (UV-2450/2550, Shimadzu). Dbait (pDNA) was used as control.

1. ***In vitro* Dbait and DOX release**

The Dbait and DOX release assays were investigated under pH-neutral (pH = 7.4), pH-acidic (pH = 5.5), and reductive acidic (pH = 5.5 + 10 mM dithiothreitol [DTT]) environment at 37°C. For Dbait release assays, the Dbait encapsulation capacity of ch-K5(s-s)R8-An/(Dbait-DOX) micelles (nitrogen/phosphorous [N/P] ratio = 10, polymer:Dbait:DOX ratio = 5.3:1.2:1.0)was evaluated by agarose gel electrophoresis method. For DOX release assays, the DOX release profile from ch-K5(s-s)R8-An/(Dbait-DOX) micelles was measured spectrophotometrically at 480 nm. For Dbait release assays, ch-K5(s-s)R8-An/(Dbait-DOX) micelles were prepared at an N/P ratio = 10. The micelles (1.06 mg polymer) were dispersed in 100 µL of PBS at pH = 7.4 without DTT or at pH = 5.5 with or without 10 mM DTT (1 mg/mL DOX and 1.2 mg/mL Dbait). After 0, 0.5, 1, 2, 3, 4, 5, 6 h incubation, the micelles solutions from each group were analyzed by agarose gel electrophoresis to evaluate the Dbait encapsulation capacity of micelles at neutral and acidic pH (pH = 7.4 and 5.5, respectively) and in a reductive acidic environment.

To investigate *in vitro* DOX release from ch-K5(s-s)R8-An/(Dbait-DOX) micelles (N/P ratio = 10, polymer:Dbait:DOX ratio = 5.3:1.2:1.0), 2 mL of the micelles was transferred into a dialysis bag (MWCO 3,500 Da). The dialysis bag was immersed in 40 mL PBS (37 °C) containing 0.1% w/v Tween 80 at pH = 7.4 without DTT or at pH = 5.5 with or without 10 mM DTT, with stirring at a speed of 110 rpm to achieve sink conditions. The 0.1% Tween 80 was added to increase DOX solubility in PBS to prevent DOX aggregation during measurement. At selected time intervals, 200 µL of the external solution was withdrawn and replaced by the same volume of fresh PBS. The DOX concentration detected at each time point was corrected by the cumulative dilution factor at the respective time point to offset dilution effects. The concentration of released DOX was measured spectrophotometrically at 480 nm.

1. ***In vitro* micelle uptake and intracellular localization**

The cellular uptake and intracellular localization of ch-K5(s-s)R8-An/(Dbait-DOX) were analyzed by imaging the YOYO-1 labeled Dbait fluorescence (red), DOX autofluorescent (green), and the 4',6-diamidino-2-phenylindole (DAPI) labeled cell nuclei (blue) using confocal laser scanning microscopy after incubating with U251 cells for 3 h. The utility of the MMP-2 cleavable linkers in ch-K5(s-s)R8-An micelles was investigated by co-incubation with MMP-2 peptides to simulate the tumor microenvironment. U251 cells were seeded at a density of 1 × 10^5^ cells/well onto sterilized microscope coverslips placed in a 24-wells plate and cultured for 24 h. Cells were grown at standard culture conditions described in the previous “Cell lines and animals” section. Prior to the preparation of the micelles/(Dbait-DOX) (0.5 μg/mL DOX and 0.6 μg/mL Dbait), Dbait was labeled with YOYO-1 for 30 min. The cells were treated with free DOX, ch-K5(s-s)R8/(Dbait/YOYO-1-DOX) micelles, ch-K5(s-s)R8-An/(Dbait/YOYO-1-DOX) micelles, or ch-K5(s-s)R8-An/(Dbait/YOYO-1-DOX) micelles in the presence of 10 pmol MMP-2 (Peprotech) and incubated for 3 h at standard culture conditions. Afterward, the cells were washed once with PBS, fixed in 4% paraformaldehyde, stained with 1 μL of DAPI (5 mg/mL), mounted, and imaged using a confocal laser scanning microscope (TCS SP8, Leica, Wetzlar, Germany) at 355/465 nm (DAPI), 491/509 nm (YOYO-1), 480/580 nm (DOX).

1. ***In vitro* cytotoxicity and anti-proliferation effect**

Cell Counting Kit-8 (CCK-8) assays were used to determine the cytotoxicity of different treatments. U251 cells were seeded in 96-well plates at a density of 8 × 10^3^ cells/well and maintained in 100 μL DMEM/well containing 10% FBS and 10 pmol of MMP-2. After 24 h, cells were treated with free DOX, ch-K5(s-s)R8-An/DOX, and ch-K5(s-s)R8-An/(Dbait-DOX) (0-4 μg/mL DOX) as well as empty micelles (ch-K5(s-s)R8-An; 0-100 μg/mL polymer). After 6 h, the cells that had been treated with ch-K5(s-s)R8-An/(Dbait-DOX) were irradiated with 2 Gy (0.3 Gy/min). After 48 h, cell viability was determined by the CCK-8 assay according to the manufacturer's protocol. Briefly, 10 μL of CCK-8 solution was added to each well and incubated for 1 h under standard culture conditions. A microplate reader (Thermo Fisher Scientific) was used to measure absorbance at 450 nm. Data were fitted in GraphPad Prism (GraphPad Software, San Diego, CA, USA) to calculate the half maximal inhibitory concentrations (IC_50_) of each treatment.

Next, the cytotoxicity of ch-K5(s-s)R8-An, DOX, Dbait + RT, ch-K5(s-s)R8-An/DOX, ch-K5(s-s)R8-An/Dbait + RT, and ch-K5(s-s)R8-An/(Dbait-DOX) + RT was assessed. U251 cells were seeded in 96-well plates at a density of 8 × 10^3^ cells/well and maintained in 100 μL DMEM/well supplemented with 10% FBS and 10 pmol of MMP-2. After 24 h, cells were treated with ch-K5(s-s)R8-An, DOX, Dbait, ch-K5(s-s)R8-An/DOX, ch-K5(s-s)R8-An/Dbait, and ch-K5(s-s)R8-An/(Dbait-DOX) (0.5 μg/mL DOX and 0.6 μg/mL Dbait). After 6 h, the cells that had been treated with Dbait were irradiated with 2 Gy (0.3 Gy/min). After 48 h, cell viability was determined by CCK-8 assay as described above.

Then, colony formation assays were performed to determine the anti-proliferation effect of various treatments.^3^ U251 cells were seeded into 6-wells plates at a density of 400 cells in 2 mL/well and cultured in DMEM containing 10% FBS and 10 pmol of MMP-2. After 24 h, cells were treated with ch-K5(s-s)R8-An, DOX, Dbait, ch-K5(s-s)R8-An/DOX, ch-K5(s-s)R8-An/Dbait, and ch-K5(s-s)R8-An/(Dbait-DOX) (0.5 μg/mL DOX and 0.6 μg/mL Dbait). After 6 h, the cells that had been treated with Dbait were irradiated with 2 Gy (0.3 Gy/min). After 14 days, cell clones were fixed with ice-cold acetic acid, incubated for 15 min at room temperature, and stained with crystal violet. All colonies with over 50 cells were counted and data were analyzed using GraphPad Prism. Untreated cells were used as control. The plating efficiency was calculated by dividing the number of colonies by the number of cells plated.

1. ***In vitro* apoptosis assay**

To determine the rate of apoptosis, U251 cells were seeded in 12-well plates at a density of 1.5 × 10^5^ cells/well and cultured in DMEM with 10% FBS and 10 pmol of MMP-2. After 24 h, cells were treated with ch-K5(s-s)R8-An, DOX, Dbait, ch-K5(s-s)R8-An/DOX, ch-K5(s-s)R8-An/Dbait, and ch-K5(s-s)R8-An/(Dbait-DOX) (0.5 μg/mL DOX and 0.6 μg/mL Dbait). After 6 h, cells that had been treated with Dbait were irradiated with 2 Gy (0.3 Gy/min). After 48 h, An annexin V-APC/propidium iodide (PI) apoptosis detection kit (BD Life Sciences, Franklin Lakes, NJ, USA) was used to determine the rate of apoptosis according to the manufacturer's protocol. Briefly, the cells were washed twice with ice-cold PBS, trypsinized, centrifuged (2,000 × g, 3 min) to gather cell pellet, and resuspended in ice-cold binding buffer. The cells were stained with annexin V-APC and propidium iodide (PI) in the dark for 10 min and analyzed by a flow cytometer (FACSCalibur; BD Biosciences, Franklin Lakes, NJ, USA). At least 10,000 events were counted in the gated region. Untreated cells were used as control. The tests were carried out in triplicate and data were processed in FlowJo software (BD Life Sciences, Franklin Lakes, NJ, USA).

1. ***In vitro* DNA damage and repair**

Western blot assays were performed to determine the DNA damage and repair levels through the expression levels of nuclear phosphorylation of histone H2A (*γ*-H_2_AX), phospho-P53 (p-P53), and DNA-dependent protein kinase catalytic subunit (DNA-PKcs). U251 cells were seeded and treated as described in the “*In vitro* Apoptosis assay” section. After 48 h, cells were harvested following trypsinization and centrifugation, then resuspended in RIPA lysis buffer containing Protease and Phosphatase Inhibitor Cocktail (Abcam, Cambridge, UK), followed by incubation on ice for 30 min. The lysates were centrifuged at 16,100 × g (Sorvall ST40, Thermo Fisher Scientific) for 10 min at 4 °C, and the supernatant was assayed for protein concentrations using the BCA protein assay kit (Thermo Fisher Scientific). Proteins were separated by SDS-PAGE (10% acrylamide, 5% SDS, 2 μg/μL protein concentration, 10 µL/well loading, run voltage 80 V for 20 min, then 120 V for 90 min), electro-transferred to polyvinylidene fluoride (PVDF) membrane (Millipore, Burlington, MA, USA; 250 V, 60 min, 4 °C), and blocked using 5% non-fat dried milk at room temperature for 1 h. The membranes were incubated overnight at 4 °C with primary antibodies (1 µg/mL; 2 µL) raised against *γ*-H_2_AX (rabbit anti-human, Abcam), DNA-PKcs (rabbit anti-human, bioWORLD, Dublin, OH, USA), p-p53 (rabbit anti-human, Abcam), and GAPDH antibody (rabbit anti-human, bioWORLD). After washing, the membranes were then incubated with HRP-conjugated secondary antibodies (Cell Signaling, Danvers, MA, USA) for 1 h at room temperature. Visualization and densitometric analysis were performed as previously described. ^4^

1. ***In vivo* brain targeting and biodistribution**

Near-infrared fluorescent boron dipyrromethene (BODIPY ,Thermo Fisher Scientific) was used to investigate the *in vivo* brain targeting ability and biodistribution property of the ch-K5(s-s)R8-An micelles. BODIPY was loaded into micelles as described in the “Preparation of micelles” section.

U251 tumor-bearing mice were constructed by implanting U251 cells into the brain tissue of nude mice. Then, and the U251 tumor-bearing mice were randomly divided into 3 groupsand were injected with free BODIPY, ch-K5(s-s)R8/BODIPY micelles, or ch-K5(s-s)R8-An/BODIPY through tail veins. After injection, the biodistribution properties were evaluated through *in vivo* fluorescence imaging and the brain tumor-targeting ability was assessed by imaging the frozen sections of brain tissues using confocal laser scanning microscopy. See Supplementary Material for details.

Male nude mice were randomly divided into three groups. Each mouse was anesthetized with 1% sodium pentobarbital and placed in a stereotaxic apparatus. U251 cells (1 × 10^5^ cells/5-μL injection volume) were then implanted into the right brain of each mouse (1 mm lateral to bregma and 3.0 mm deep from the dura) using a microvolume injector (Hamilton, Reno, NV, USA). After 14 d, *In situ* U251 tumor-bearing mice were injected intravenously through the tail vein with 200 μL of BODIPY, ch-K5(s-s)R8/BODIPY, or ch-K5(s-s)R8-An/BODIPY at a dose of 5 μg BODIPY per mouse. After injection, the biodistribution property of micelles was evaluated through in vivo fluorescence imaging (Xenogen IVIS-200, Caliper Life Sciences, Hopkinton, MA, USA) at 646/660 nm with 3 s exposure time. To investigate the brain tumor-targeting ability of micelles, the brain was dissected from U251 tumor-bearing mice 4 h after intravenous injection. 20 µm frozen sections of brain tissues were cut with a cryotome (RM2016, Leica), stained with 1 μL of DAPI (5 mg/mL), and imaged using a confocal laser scanning microscope (TCS SP8, Leica) at 646/660 nm.

1. ***In vivo* anti-tumor efficacy**

To evaluate the collaborative therapeutic effect of ch-K5(s-s)R8-An/(Dbait-DOX) micelles plus RT, *in situ* U251 tumor-bearing mice were randomly allocated to seven groups (n = 12/group): control, free DOX, RT, ch-K5(s-s)R8-An/DOX micelles, ch-K5(s-s)R8-An/Dbait micelles + RT, ch-K5(s-s)R8/DOX micelles + RT, and ch-K5(s-s)R8-An/(Dbait-DOX) micelles + RT. Intracranial xenografting of U251 cells to establish an orthotopic glioblastoma model was performed as described in the “*In vivo* biodistribution” section.

Accordingly, micelles, free DOX, vehicle control or physiological saline control were intravenously administered on day 12, 19, and 26 after xenografting (polymer, 10.6 mg/kg; DOX, 2 mg/kg; Dbait, 2.4 mg/kg) through the tail vein. The injection volume (V) was calculated as: V = (2 mg/kg × body weight) / the concentration of DOX. The DOX concentration was 1 mg/mL and the Dbait concentration was 1.2 mg/mL. RT was administered on day 13, 20, and 27 at a cumulative dose of 2 Gy (0.3 Gy/min) at 24 h after injection of the different formulations. Local RT was performed by focusing the X-rays (electron linear accelerator, Varian, Palo Alto, CA) into the right brain. 24 h after the last RT treatment, intracranial tumors were imaged by Magnetic resonance imaging (MRI, Magnetom Aera, Siemens, Munich, Germany) on day 28 after U251 cell inoculation.

Then, part of the animals were sacrificed (n = 6/group) to collect major organs (heart, liver, spleen, lung, kidney, brain), and the rest animals were kept alive under standard care conditions. Body weights of living mice were recorded every two days and mice survival was monitored to construct Kaplan-Meier curves (n = 6/group). These dissected organs were rinsed with PBS, fixed in 4% paraformaldehyde (Sangon Biotech) for 24 h at room temperature, embedded in paraffin, and cut into 5 μm sections. The histological damage levels of the brain tumor sections were assessed by hematoxylin and eosin (H&E) staining and *γ*-H_2_AX immunofluorescence staining. Meanwhile, the *in vivo* safety of each treatment was evaluated by the H&E histological staining of organ sections, which was performed with H&E Staining Kit (Abcam) following standard protocol and imaged under a light microscope (DMIL, Leica, Wetzlar, Germany). Immunohistochemical staining of *γ*-H_2_AX in tumor tissue was performed on 5 μm paraffin sections as described. ^5^ Briefly, paraffin sections of tumor tissue were dewaxed at 65 °C for 4 h followed by two 15 min washes with xylene and then rehydrated with graded ethanol and distilled water. Antigen retrieval was carried out by heating the sections in 10 mM citrate buffer (pH = 6.0) at 95 °C for 30 min, and endogenous peroxidases were blocked by hydrogen peroxide blocking reagent (Abcam) for 10 min. After 30 min blocking with 5% bovine serum albumin (Thermo Fisher Scientific), the sections were washed three times with PBS and incubated overnight at 4 °C with 5 µg/mL rabbit anti-human γ-H2AX (Abcam). Next, the sections were incubated with Alexa Fluor 488-conjugated secondary goat anti-rabbit IgG antibodies for 30 min at room temperature, then stained with 1 μL of DAPI (5 mg/mL). Finally, the sections were dehydrated, sealed with coverslips, and imaged using a confocal laser scanning microscope (TCS SP8, Leica, Wetzlar, Germany) at 495/519 nm.

1. **Statistical analyses**

All treatments were repeated in triplicate and all values were presented as the means ± standard deviations (SD). Statistical analysis was performed using the one-way analysis of variance (ANOVA). The difference was considered as significant when the *P-*value was less than 0.05.

**Table S1.** Particle characterization of different micelle formulations.

|  | ch-K5(s-s)R8-An/ DOX | ch-K5(s-s)R8-An/ Dbait | ch-K5(s-s)R8-An/ (Dbait-DOX) |
| --- | --- | --- | --- |
| Mean size^a^ [nm] | 187 ± 4 | 128 ± 6 | 142 ± 2 |
| PDI^a^ | 0.186 ± 0.021 | 0.268 ± 0.049 | 0.219 ± 0.052 |
| Zeta potential^b^ [mV] | 31.6 ± 1.6 | 21.2 ± 1.6 | 20.2 ± 1.3 |

Micelles were prepared at the following component concentrations: polymer, 5.3 mg/mL; Dbait, 1.2 mg/mL; DOX: 1.0 mg/mL, yielding a polymer:Dbait:DOX ratio of 5.3:1.2:1.0.

^a^ Mean hydrodynamic diameter measured in PBS (pH = 7.4) at 25 °C

^b^ Measured in PBS (pH = 7.4) at 25 °C

Data are shown as mean ± SD (*n* = 3)

**Table S2.** Median survival time of tumor-bearing mice after different treatments

| Group | Median (days) | Increased survival rate (%) |
| --- | --- | --- |
| Control | 26 | --- |
| DOX | 32 | 23.08% |
| RT | 33 | 26.92% |
| ch-K5(s-s)R8-An/DOX ^a, c^ | 40.5 | 55.77% |
| ch-K5(s-s)R8-An/Dbait+RT ^a, d^ | 39.5 | 51.92% |
| ch-K5(s-s)R8/(Dbait-DOX)+RT ^b, c, d^ | 44 | 69.23% |
| ch-K5(s-s)R8-An/(Dbait-DOX)+RT ^b, c, d,e^ | 56 | 115.38% |

^a^*p*<0.05 *vs* control, ^b^*p*<0.001 *vs* control, ^c^*p*<0.05 *vs* DOX, ^d^*p*<0.05 *vs* RT, ^e^*p*<0.05 *vs* ch-K5(s-s)R8/(Dbait-DOX)+RT

**References**

1. Koo AN, Min KH, Lee HJ, et al. Tumor accumulation and antitumor efficacy of docetaxel-loaded core-shell-corona micelles with shell-specific redox-responsive cross-links. *Biomaterials*. Feb 2012;33(5):1489-99. doi:10.1016/j.biomaterials.2011.11.013

2. Shao K, Zhang Y, Ding N, et al. Functionalized nanoscale micelles with brain targeting ability and intercellular microenvironment biosensitivity for anti-intracranial infection applications. *Adv Healthc Mater*. Jan 28 2015;4(2):291-300. doi:10.1002/adhm.201400214

3. Ding B, Wahid MA, Wang Z, et al. Triptolide and celastrol loaded silk fibroin nanoparticles show synergistic effect against human pancreatic cancer cells. *Nanoscale*. Aug 17 2017;9(32):11739-11753. doi:10.1039/c7nr03016a

4. Jiao X, Yu Y, Meng J, et al. Dual-targeting and microenvironment-responsive micelles as a gene delivery system to improve the sensitivity of glioma to radiotherapy. *Acta Pharm Sin B*. Mar 2019;9(2):381-396. doi:10.1016/j.apsb.2018.12.001

5. Yao H, Qiu H, Shao Z, et al. Nanoparticle formulation of small DNA molecules, Dbait, improves the sensitivity of hormone-independent prostate cancer to radiotherapy. *Nanomedicine*. Nov 2016;12(8):2261-2271. doi:10.1016/j.nano.2016.06.010
